# Chlorimipraminium picrate

**DOI:** 10.1107/S1600536810000905

**Published:** 2010-01-13

**Authors:** Jerry P. Jasinski, Ray J. Butcher, Q. N. M. Hakim Al-Arique, H. S. Yathirajan, B. Narayana

**Affiliations:** a Department of Chemistry, Keene State College, 229 Main Street, Keene, NH 03435-2001, USA; b Department of Chemistry, Howard University, 525 College Street NW, Washington, DC 20059, USA; c Department of Studies in Chemistry, University of Mysore, Manasagangotri, Mysore 570 006, India; d Department of Studies in Chemistry, Mangalore University, Mangalagangotri 574 199, India

## Abstract

The title compound {systematic name: 3-chloro-5-[3-(dimethyl­amino)prop­yl]-10,11-dihydro-5*H*-dibenz[*b*,*f*]azepinium picrate}, C_19_H_24_ClN_2_^+^·C_6_H_2_N_3_O_7_^−^, crystallizes with two independent cation–anion pairs in the asymmetric unit. The chlorimipraminium cation contains two benzene rings (one with a chloro substituent) fused to a V-shaped seven-membered azepine ring whose mean planes are separated by 61.1 (0) and 66.5 (8)° with a 3-(dimethyl­amino)propyl group extending away from the apex of this ring. In the picrate anion, the mean planes of the two *o*-NO_2_ groups in each anion are twisted by 3.7 (2)/31.9 (3) and 31.3 (1)/11.4 (0)°, respectively, with respect to the mean plane of the six-membered benzene ring. The phenolate O atoms are bent slightly away from the mean plane of the benzene ring. The mean planes of the *p*-NO_2_ groups are twisted by 6.6 (1) and 2.88°, respectively, from the mean plane of the benzene ring. The crystal packing features bifurcated N—H⋯(O,O) inter­molecular hydrogen-bond inter­action, which connects each cation–anion pair. Additional π–π ring and C—H⋯π weak inter­molecular inter­actions are also observed.

## Related literature

For related structures, see: Bindya *et al.* (2007 ▶); Hallberg *et al.* (1984 ▶); Harrison, Bindya *et al.* (2007 ▶); Hallberg *et al.* (1984 ▶); Harrison, Sreevidya *et al.* (2007 ▶); Post *et al.* (1975 ▶); Post & Horn (1977 ▶); Swamy *et al.* (2007 ▶); Yathirajan *et al.* (2007 ▶). For obessive-compulsive disorder treatment, see: Albert *et al.* (2002 ▶). For pain disorder treatment, see: Cassano *et al.* (1988 ▶). For non-toxic cancer-therapeutic activity, see: Daley *et al.* (2005 ▶). For experimental anxiety in humans, see: Guimaraes *et al.* (1987 ▶). For quantum mechanical calculations, see: Becke (1988 ▶); Schmidt & Polik (2007 ▶); Frisch *et al.* (2004 ▶); Lee *et al.* (1988 ▶).
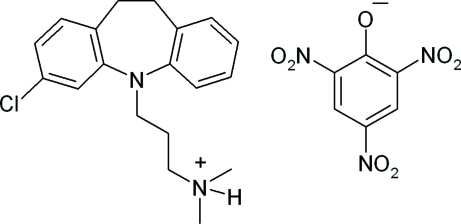


## Experimental

### 

#### Crystal data

C_19_H_24_ClN_2_^+^·C_6_H_2_N_3_O_7_^−^
*M* *_r_* = 543.96
Triclinic, 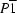

*a* = 11.2252 (3) Å
*b* = 13.1514 (3) Å
*c* = 17.2787 (4) Å
α = 90.9414 (19)°
β = 91.1253 (19)°
γ = 100.4446 (19)°
*V* = 2507.55 (10) Å^3^
*Z* = 4
Mo *K*α radiation
μ = 0.21 mm^−1^
*T* = 110 K
0.47 × 0.41 × 0.15 mm

#### Data collection

Oxford Diffraction Gemini R CCD diffractometer
Absorption correction: multi-scan (*CrysAlis RED*; Oxford Diffraction, 2007 ▶) *T*_min_ = 0.918, *T*_max_ = 0.969
32779 measured reflections
16481 independent reflections
10873 reflections with *I* > 2σ(*I*)
*R*_int_ = 0.023

#### Refinement

*R*[*F* ^2^ > 2σ(*F* ^2^)] = 0.044
*wR*(*F* ^2^) = 0.115
*S* = 0.98
16481 reflections
689 parameters
H-atom parameters constrained
Δρ_max_ = 0.53 e Å^−3^
Δρ_min_ = −0.48 e Å^−3^

### 

Data collection: *CrysAlis PRO* (Oxford Diffraction, 2007 ▶); cell refinement: *CrysAlis RED* (Oxford Diffraction, 2007 ▶); data reduction: *CrysAlis RED*; program(s) used to solve structure: *SHELXS97* (Sheldrick, 2008 ▶); program(s) used to refine structure: *SHELXL97* (Sheldrick, 2008 ▶); molecular graphics: *XP* (Sheldrick, 2008 ▶) and *Mercury* (Macrae *et al.*, 2006 ▶); software used to prepare material for publication: *SHELXTL* (Sheldrick, 2008 ▶), *enCIFer* (Allen *et al.*, 2004 ▶) and *PLATON* (Spek, 2009 ▶).

## Supplementary Material

Crystal structure: contains datablocks global, I. DOI: 10.1107/S1600536810000905/bt5128sup1.cif

Structure factors: contains datablocks I. DOI: 10.1107/S1600536810000905/bt5128Isup2.hkl

Additional supplementary materials:  crystallographic information; 3D view; checkCIF report

## Figures and Tables

**Table 1:** Hydrogen-bond geometry (Å, °)

*D*—H⋯*A*	*D*—H	H⋯*A*	*D*⋯*A*	*D*—H⋯*A*
N2*A*—H2*AB*⋯O1*D*	0.93	1.85	2.7000 (13)	151
N2*A*—H2*AB*⋯O21*D*	0.93	2.23	2.8982 (14)	128
N2*B*—H2*BB*⋯O1*C*	0.93	1.92	2.6970 (13)	140
N2*B*—H2*BB*⋯O62*C*	0.93	2.36	3.0657 (15)	133
C12*A*—H12*A*⋯*Cg*7^i^	0.95	2.83	3.656 (4)	145

Symmetry code: (i) 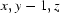. *Cg*7 is the centroid of the C1*B*–C6*B* ring.

**Table 2:** π-π hydrogen-bond geometry (Å)

*Cg*⋯*Cg*	D⋯A
*Cg*1⋯*Cg*14^i^	3.838 (8)
*Cg*7⋯*Cg*13^ii^	3.473 (5)
*Cg*13⋯*Cg*14^i^	3.590 (5)

Symmetry codes: (i) *x*, *y*, *z*; (ii) 1 − *x*, *y*, *z*; *Cg*1, *Cg*7, *Cg*13 and *Cg*14 are the centroids of the C1*A*–C6*A*, C1*B*–C6*B*, C1*C*–C6*C* and C1*D*–C6*D* rings.

**Fig. 1.**
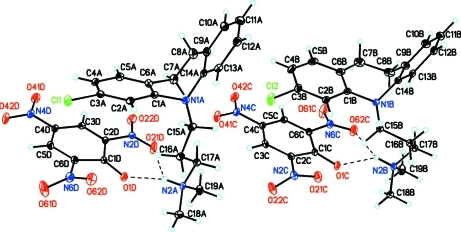
Molecular structure of the two C19H24ClN2+. C6H2N3O7- cation-anion pairs in the asymmetric unit showing the atom labeling scheme and 50% probability displacement ellipsoids. Dashed lines indicate bifurcated (3-center) N2A—H2AB···O1D, N2A—H2AB···O2D and N2B—H2BB···O1C, N2B—H2BB···O62C, cation-anion hydrogen bond interactions.

**Fig. 2.**
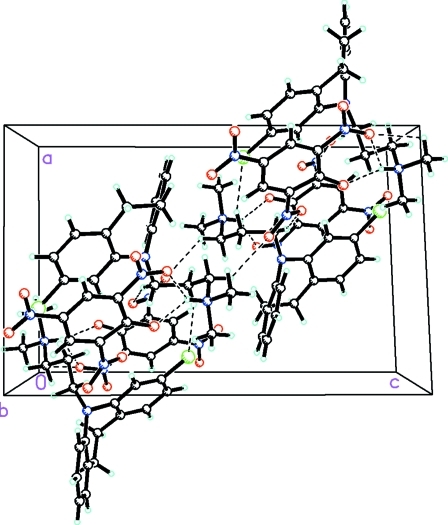
Packing diagram of the title compound, (I), viewed down the b axis. Dashed lines indicate intermolecular N—H···O hydrogen bond interactions which produces a two-dimensional network of infinite O—H···O—H···O—H chains arranged along the (101) plane of the unit cell.

**Fig. 3.**
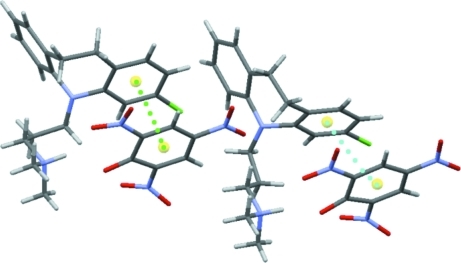
π-π interactions in the asymmetric of (I).

## supplementary crystallographic information

### Comment

Chlorimipramine [IUPAC name: 3-chloro-5-(3-dimethylaminopropyl)-10, 11-dihydro-5*H*-dibenz[b,f]azepine] is a tricyclic antidepressant that was developed in the 1960's by the Swiss drug manufacturer Geigy (now known as Novartis) and has been in clinical use worldwide for decades. Chlorimipramine, a 3-chloro derivative of imipramine, is a strong, but not completely selective, serotonin reuptake inhibitor (SSRI), which like the primary active metabolite desmethylclomipramine acts preferably as a norepinephrine reuptake inhibitor. It is used in the treatment of obsessive-compulsive disorder (Albert *et al.*, 2002) and panic disorder (Cassano *et al.*, 1988). The effect of chlorimipramine and maprotiline on experimental anxiety in humans has been reported (Guimaraes *et al.*, 1987). The use of chlorimipramine in humans as an effective, non-toxic cancer-therapeutic having a strong selectivity between cancer cells and normal cells on the basis of their mitochondrial function has also been discussed (Daley *et al.*, 2005).

The crystal and molecular structure of tricyclic antidepressant imipramine hydrochloride (Post *et al.*, 1975), chloripramine hydrochloride (Post & Horn, 1977; Hallberg *et al.*, 1984), amitriptylinium picrate (Bindya *et al.*, 2007), mepazinium picrate (Yathirajan *et al.*, 2007), imipraminium picrate (Harrison, Bindya *et al.*, 2007), nevirapinium picrate (Harrison, Sreevidya *et al.*, 2007) and desipraminium picrate (Swamy *et al.*, 2007) have been reported. In view of the importance of chlorimipramine and to study the hydrogen bonding patterns in the title compound, (I), C_25_H_26_ClN_5_O_7_, a crystal structure is reported.

The title compound,*C*_25_H_26_ClN_5_O_7_, crystallizes with two independent cation-anion pairs [C_19_H_24_ClN_2_^+^. C_6_H_2_N_3_O_7_^-^] in the asymmetric unit (Fig. 1). The chlorimipraminium cation contains two benzene rings (one halogenated) fused to a V-shaped, seven-membered azepine ring whose mean planes are separated by 61.1 (0)° (A) and 66.5 (8)° (B) with a 3-dimethylaminopropyl) group extending away from the apex of the bent azepine group (Torsion angles C1A—N1A—C15A—C16A = 59.19 (14)°; C1B—N1B—C15B—C16B = 165.18 (10)°). In the picrate anion, the mean planes of the two *o*-NO_2_ groups (O21D—N2D—O22D & O61D—N6D—O62D; O21C—N2C—O22C & O61C—N6C—O62C are twisted by 3.7 (2)°, 31.9 (3)° and 31.3 (1)°, 11.4 (0)°, respectively, with respect to the mean plane of the 6-membered benzene ring (Fig. 1). The phenolate oxygen atoms are bent slightly away from the mean plane of the benzene ring (Torsion angles O1D—C1D—C2D—C3D = 171.41 (12)°; O1C—C1C—C2C—C3C = -172.94 (12)°). The mean planes of the *p*-NO_2_ oxygen atoms (O41D—N4D—O42D & O41C—N4C—O42C) are twisted by 6.6 (1)° and 2.88°, respectively, from the mean plane of the benzene ring. The difference in the twist angles of the mean planes of the two *o*-NO_2_ groups and the bend in the phenolate oxygen atoms in each of the cation units can be attributed to the influence of strong bifurcated (3-center) "side" hydrogen bond intermolecular interactions with the nitrogen atom in the 3-dimethylaminopropyl group of their cation neighbors (N2A—H2AB···O1D, N2A—H2AB···O2D and N2B—H2BB···O1C, N2B—H2BB···O62C; Fig. 2, Table 1). Crystal packing is also influenced by additional weak π–π ring intermolecular interactions (Table 2, Fig. 3) and a weak C12A—H12A···*Cg*7 π-ring intermoleclar interaction (H12A···*Cg*7 = 2.83°, C12*a*—H12A···*Cg*7 = 145°, C12A···*Cg*7 = 3.656 (4)°; *x*, -1 + *y*, *z*).

A density functional theory (DFT) geometry optimization molecular orbital calculation (Schmidt & Polik, 2007) was performed on the two independent cation-anion pairs (C_19_H_24_ClN_2_^+^. C_6_H_2_N_3_O_7_^-^) of the asymmetric unit with the *GAUSSIAN03* program package (Frisch *et al.*, 2004; Becke, 1988; Lee *et al.*, 1988) and the 3–21 G basis set. Starting geometries were taken from X-ray refinement data. In the cation, the angle between the two benzene rings fused to the azepine ring decreases to 53.2 (9)° (A) and 55.8 (5)°, a change of -7.8 (1)° and -10.7 (3)°, respectively. In the picrate anion, the mean planes of the two *o*-NO_2_ groups (O21D—N2D—O22D & O61D—N6D—O62D; O21C—N2C—O22C & O61C—N6C—O62C become twisted by 18.2 (8)°, 6.3 (5)° and 15.5 (6)°, 13.6 (4)°, with respect to the mean plane of the 6-membered benzene ring, changes of +14.5 (6)°, -25.5 (8)° and -15.7 (5)°, +2.2 (4)°, respectively. The torsion angles of the phenolate oxygen atoms (O1D—C1D—C2D—C3D & O1C—C1C—C2C—C3C) become 175.1 (6)° and -171.6 (7)°, changes of +3.7 (5)° and -1.2 (7)° relative to the mean plane of the benzene ring. The mean planes of the *p*-NO_2_ oxygen atoms (O41D—N4D—O42D & O41C—N4C—O42C) become twisted by 0.4 (8)° and 6.2 (3)°, changes of -6.3 (1)° and +3.3 (5)°, respectively, from the mean plane of the anion benzene ring. The dihedral angle between the benzene ring of the anion and the benzene and chloro substituted benzene ring in the cation change from 70.1 (5)°, 15.0 (4)° and 67.9 (3)°, 4.3 (5)° to 35.1 (5)°, 44.8 (3)° and 36.7 (3)°, 12.0 (2)°, changes of -35.0 (0)°, +21.6 (9)°, and -23.1 (0)°, +7.6 (7)°, respectively. Examination of the partial charges from the DFT geometry optimization indicate that H2BD (0.43570) is slightly more positive than H2AB (0.424847) producing a slightly stronger proton charge associated with the N2B atom of the cation-anion pair (B & C groups).

### Experimental

Clorimipramine hydrochloride (3.5 g, 0.01 mol) in 25 ml of a mixture of (1:1) acetonitrile & methanol and picric acid (4.5 g, 0.01 mol) in 25 ml of mixture of (1:1) acetonitrile & methanol were mixed and stirred in a beaker at 318 K for two hours. The mixture was kept aside for about five days at room temperature. The separated salt was filtered and dried in vacuum desiccator over phosphorous pentoxide. The crystals obtained (m.p: 393 - 395 K) were used for *x*-ray studies.

### Refinement

All of the H atoms were placed in their calculated positions and then refined using the riding model with N—H = 0.93 Å, C—H = 0.95–0.99 Å, and with *U*_iso_(H) = 1.18–1.51*U*_eq_(C,*N*).

### Crystal data

**Table:**

C_19_H_24_ClN_2_^+^·C_6_H_2_N_3_O_7_^−^	*Z* = 4
*M**_r_* = 543.96	*F*(000) = 1136
Triclinic, *P*1	*D*_x_ = 1.441 Mg m^−^^3^
Hall symbol: -P 1	Mo *K*α radiation, λ = 0.71073 Å
*a* = 11.2252 (3) Å	Cell parameters from 14780 reflections
*b* = 13.1514 (3) Å	θ = 4.7–32.7°
*c* = 17.2787 (4) Å	µ = 0.21 mm^−^^1^
α = 90.9414 (19)°	*T* = 110 K
β = 91.1253 (19)°	Plate, yellow
γ = 100.4446 (19)°	0.47 × 0.41 × 0.15 mm
*V* = 2507.55 (10) Å^3^	

### Data collection

**Table:**

Oxford Diffraction Gemini R CCD diffractometer	16481 independent reflections
Radiation source: Enhance (Mo) X-ray Source	10873 reflections with *I* > 2σ(*I*)
graphite	*R*_int_ = 0.023
Detector resolution: 10.5081 pixels mm^-1^	θ_max_ = 32.8°, θ_min_ = 4.7°
φ and ω scans	*h* = −16→16
Absorption correction: multi-scan (*CrysAlis RED*; Oxford Diffraction, 2007)	*k* = −19→16
*T*_min_ = 0.918, *T*_max_ = 0.969	*l* = −23→26
32779 measured reflections	

### Refinement

**Table:**

Refinement on *F*^2^	Primary atom site location: structure-invariant direct methods
Least-squares matrix: full	Secondary atom site location: difference Fourier map
*R*[*F*^2^ > 2σ(*F*^2^)] = 0.044	Hydrogen site location: inferred from neighbouring sites
*wR*(*F*^2^) = 0.115	H-atom parameters constrained
*S* = 0.98	*w* = 1/[σ^2^(*F*_o_^2^) + (0.0635*P*)^2^] where *P* = (*F*_o_^2^ + 2*F*_c_^2^)/3
16481 reflections	(Δ/σ)_max_ < 0.001
689 parameters	Δρ_max_ = 0.53 e Å^−^^3^
0 restraints	Δρ_min_ = −0.48 e Å^−^^3^

### Special details

**Table:**

Geometry. All e.s.d.'s (except the e.s.d. in the dihedral angle between two l.s. planes) are estimated using the full covariance matrix. The cell e.s.d.'s are taken into account individually in the estimation of e.s.d.'s in distances, angles and torsion angles; correlations between e.s.d.'s in cell parameters are only used when they are defined by crystal symmetry. An approximate (isotropic) treatment of cell e.s.d.'s is used for estimating e.s.d.'s involving l.s. planes.
Refinement. Refinement of *F*^2^ against ALL reflections. The weighted *R*-factor *wR* and goodness of fit *S* are based on *F*^2^, conventional *R*-factors *R* are based on *F*, with *F* set to zero for negative *F*^2^. The threshold expression of *F*^2^ > σ(*F*^2^) is used only for calculating *R*-factors(gt) *etc*. and is not relevant to the choice of reflections for refinement. *R*-factors based on *F*^2^ are statistically about twice as large as those based on *F*, and *R*- factors based on ALL data will be even larger.

### Fractional atomic coordinates and isotropic or equivalent isotropic displacement parameters (Å^2^)

**Table:**

	*x*	*y*	*z*	*U*_iso_*/*U*_eq_	
Cl1	0.59334 (3)	0.87280 (3)	0.92251 (2)	0.03074 (9)	
N1A	0.37421 (9)	0.86170 (8)	0.65507 (6)	0.0172 (2)	
N2A	0.64917 (9)	0.73998 (8)	0.53424 (6)	0.0175 (2)	
H2AB	0.6526	0.7035	0.5796	0.021*	
C1A	0.39922 (10)	0.81199 (9)	0.72519 (7)	0.0166 (2)	
C2A	0.47865 (10)	0.86207 (10)	0.78235 (7)	0.0183 (2)	
H2AA	0.5199	0.9309	0.7755	0.022*	
C3A	0.49670 (11)	0.81002 (10)	0.84951 (7)	0.0198 (3)	
C4A	0.43734 (11)	0.71005 (10)	0.86195 (7)	0.0200 (3)	
H4AA	0.4498	0.6760	0.9087	0.024*	
C5A	0.35871 (11)	0.66092 (10)	0.80371 (8)	0.0210 (3)	
H5AA	0.3167	0.5925	0.8114	0.025*	
C6A	0.34025 (11)	0.70941 (10)	0.73483 (7)	0.0184 (2)	
C7A	0.25976 (12)	0.65622 (10)	0.67027 (8)	0.0240 (3)	
H7AA	0.2404	0.5812	0.6802	0.029*	
H7AB	0.3038	0.6655	0.6211	0.029*	
C8A	0.14237 (12)	0.69788 (11)	0.66183 (9)	0.0288 (3)	
H8AA	0.0972	0.6631	0.6160	0.035*	
H8AB	0.0931	0.6765	0.7077	0.035*	
C9A	0.14981 (11)	0.81328 (10)	0.65337 (7)	0.0212 (3)	
C10A	0.03832 (12)	0.84621 (11)	0.64822 (8)	0.0265 (3)	
H10A	−0.0339	0.7961	0.6512	0.032*	
C11A	0.02888 (12)	0.94905 (12)	0.63898 (8)	0.0273 (3)	
H11A	−0.0484	0.9685	0.6342	0.033*	
C12A	0.13302 (12)	1.02253 (11)	0.63690 (8)	0.0246 (3)	
H12A	0.1281	1.0934	0.6314	0.030*	
C13A	0.24533 (11)	0.99277 (10)	0.64291 (7)	0.0201 (3)	
H13A	0.3168	1.0440	0.6420	0.024*	
C14A	0.25533 (11)	0.88889 (10)	0.65020 (7)	0.0171 (2)	
C15A	0.47414 (11)	0.93650 (10)	0.62436 (8)	0.0192 (2)	
H15A	0.4982	0.9939	0.6625	0.023*	
H15B	0.4462	0.9658	0.5763	0.023*	
C16A	0.58398 (11)	0.88708 (10)	0.60687 (7)	0.0189 (2)	
H16A	0.6517	0.9410	0.5906	0.023*	
H16B	0.6103	0.8554	0.6543	0.023*	
C17A	0.55383 (11)	0.80490 (10)	0.54323 (7)	0.0191 (2)	
H17A	0.4761	0.7595	0.5548	0.023*	
H17B	0.5429	0.8390	0.4936	0.023*	
C18A	0.77140 (11)	0.80309 (11)	0.52275 (8)	0.0268 (3)	
H18A	0.7975	0.8448	0.5696	0.040*	
H18B	0.7680	0.8488	0.4788	0.040*	
H18C	0.8292	0.7572	0.5122	0.040*	
C19A	0.61616 (13)	0.66325 (11)	0.46961 (7)	0.0249 (3)	
H19A	0.5368	0.6208	0.4790	0.037*	
H19B	0.6772	0.6188	0.4664	0.037*	
H19C	0.6127	0.6996	0.4208	0.037*	
Cl2	0.19710 (4)	0.83645 (3)	0.44656 (2)	0.03975 (10)	
N1B	−0.00504 (8)	0.84031 (8)	0.17770 (6)	0.0164 (2)	
N2B	0.16727 (9)	0.76659 (8)	0.02222 (6)	0.0194 (2)	
H2BB	0.1600	0.7376	0.0710	0.023*	
C1B	−0.00482 (11)	0.79100 (9)	0.25044 (7)	0.0179 (2)	
C2B	0.08559 (11)	0.82856 (10)	0.30604 (7)	0.0196 (2)	
H2BA	0.1479	0.8848	0.2941	0.024*	
C3B	0.08492 (13)	0.78428 (11)	0.37833 (8)	0.0257 (3)	
C4B	−0.00406 (14)	0.70222 (12)	0.39755 (9)	0.0322 (3)	
H4BA	−0.0046	0.6725	0.4473	0.039*	
C5B	−0.09206 (13)	0.66473 (11)	0.34232 (9)	0.0307 (3)	
H5BA	−0.1533	0.6081	0.3552	0.037*	
C6B	−0.09587 (11)	0.70576 (10)	0.26812 (9)	0.0242 (3)	
C7B	−0.19630 (13)	0.65203 (12)	0.21395 (10)	0.0354 (4)	
H7BA	−0.2743	0.6529	0.2395	0.043*	
H7BB	−0.1874	0.5787	0.2086	0.043*	
C8B	−0.20590 (12)	0.69393 (11)	0.13257 (9)	0.0299 (3)	
H8BA	−0.1331	0.6859	0.1033	0.036*	
H8BB	−0.2777	0.6536	0.1050	0.036*	
C9B	−0.21672 (11)	0.80566 (10)	0.13599 (8)	0.0224 (3)	
C10B	−0.32578 (12)	0.83958 (12)	0.12103 (8)	0.0283 (3)	
H10B	−0.3956	0.7910	0.1050	0.034*	
C11B	−0.33334 (12)	0.94331 (12)	0.12930 (8)	0.0286 (3)	
H11B	−0.4079	0.9655	0.1188	0.034*	
C12B	−0.23241 (12)	1.01431 (11)	0.15281 (7)	0.0236 (3)	
H12B	−0.2380	1.0852	0.1587	0.028*	
C13B	−0.12244 (11)	0.98269 (10)	0.16791 (7)	0.0181 (2)	
H13B	−0.0529	1.0319	0.1833	0.022*	
C14B	−0.11529 (10)	0.87815 (10)	0.16026 (7)	0.0176 (2)	
C15B	0.10829 (10)	0.90796 (9)	0.15678 (7)	0.0160 (2)	
H15C	0.1776	0.8734	0.1689	0.019*	
H15D	0.1187	0.9729	0.1880	0.019*	
C16B	0.10798 (11)	0.93317 (10)	0.07102 (7)	0.0191 (2)	
H16C	0.0473	0.9779	0.0615	0.023*	
H16D	0.1884	0.9735	0.0586	0.023*	
C17B	0.08041 (11)	0.84068 (10)	0.01625 (7)	0.0201 (3)	
H17C	−0.0022	0.8027	0.0263	0.024*	
H17D	0.0804	0.8658	−0.0375	0.024*	
C18B	0.29610 (11)	0.81780 (11)	0.01351 (8)	0.0245 (3)	
H18D	0.3482	0.7655	0.0146	0.037*	
H18E	0.3199	0.8679	0.0561	0.037*	
H18F	0.3049	0.8536	−0.0359	0.037*	
C19B	0.13262 (14)	0.68120 (11)	−0.03663 (8)	0.0293 (3)	
H19D	0.0488	0.6468	−0.0287	0.044*	
H19E	0.1868	0.6310	−0.0311	0.044*	
H19F	0.1396	0.7098	−0.0887	0.044*	
O1C	0.26076 (8)	0.70245 (7)	0.15382 (5)	0.0229 (2)	
O21C	0.38889 (9)	0.86117 (7)	0.24018 (6)	0.0298 (2)	
O22C	0.50236 (9)	0.79895 (8)	0.32264 (7)	0.0370 (3)	
O41C	0.27695 (10)	0.55178 (8)	0.49309 (5)	0.0319 (2)	
O42C	0.12957 (9)	0.43593 (8)	0.44634 (6)	0.0346 (3)	
O61C	−0.00593 (11)	0.44920 (9)	0.18672 (7)	0.0511 (4)	
O62C	0.04994 (9)	0.58148 (9)	0.11703 (6)	0.0384 (3)	
N2C	0.41012 (9)	0.79142 (8)	0.28158 (7)	0.0214 (2)	
N4C	0.20810 (10)	0.51373 (9)	0.43944 (6)	0.0229 (2)	
N6C	0.05988 (10)	0.53218 (9)	0.17559 (6)	0.0225 (2)	
C1C	0.24304 (10)	0.66219 (9)	0.21843 (7)	0.0156 (2)	
C2C	0.31920 (10)	0.69712 (9)	0.28621 (7)	0.0161 (2)	
C3C	0.31107 (11)	0.64889 (9)	0.35594 (7)	0.0174 (2)	
H3CA	0.3660	0.6739	0.3974	0.021*	
C4C	0.22078 (11)	0.56251 (9)	0.36500 (7)	0.0171 (2)	
C5C	0.14014 (10)	0.52518 (10)	0.30518 (7)	0.0175 (2)	
H5CA	0.0786	0.4665	0.3126	0.021*	
C6C	0.14961 (10)	0.57354 (9)	0.23489 (7)	0.0168 (2)	
O1D	0.71604 (8)	0.69222 (7)	0.67815 (5)	0.0226 (2)	
O21D	0.52660 (10)	0.56347 (9)	0.61786 (6)	0.0393 (3)	
O22D	0.43317 (9)	0.44269 (9)	0.68675 (6)	0.0362 (3)	
O41D	0.53373 (9)	0.39608 (8)	0.94767 (6)	0.0316 (2)	
O42D	0.68754 (10)	0.49588 (8)	1.00391 (6)	0.0322 (2)	
O61D	0.91601 (11)	0.78149 (10)	0.86413 (8)	0.0555 (4)	
O62D	0.81134 (9)	0.84577 (8)	0.77794 (6)	0.0337 (2)	
N2D	0.51250 (9)	0.51864 (8)	0.67940 (6)	0.0176 (2)	
N4D	0.61854 (10)	0.47077 (9)	0.94774 (6)	0.0232 (2)	
N6D	0.83039 (10)	0.77441 (9)	0.81814 (7)	0.0266 (3)	
C1D	0.68478 (10)	0.64639 (9)	0.73884 (7)	0.0149 (2)	
C2D	0.59071 (10)	0.55516 (9)	0.74548 (7)	0.0152 (2)	
C3D	0.56982 (10)	0.49945 (9)	0.81231 (7)	0.0167 (2)	
H3DA	0.5083	0.4395	0.8129	0.020*	
C4D	0.63868 (11)	0.53121 (10)	0.87829 (7)	0.0176 (2)	
C5D	0.72585 (11)	0.62116 (10)	0.87969 (7)	0.0198 (3)	
H5DA	0.7717	0.6434	0.9256	0.024*	
C6D	0.74406 (11)	0.67694 (9)	0.81332 (7)	0.0175 (2)	

### Atomic displacement parameters (Å^2^)

**Table:**

	*U*^11^	*U*^22^	*U*^33^	*U*^12^	*U*^13^	*U*^23^
Cl1	0.02852 (17)	0.0392 (2)	0.02166 (16)	−0.00019 (14)	−0.00720 (13)	−0.00441 (14)
N1A	0.0177 (5)	0.0158 (5)	0.0173 (5)	0.0004 (4)	0.0016 (4)	0.0029 (4)
N2A	0.0208 (5)	0.0189 (5)	0.0131 (5)	0.0044 (4)	−0.0010 (4)	0.0026 (4)
C1A	0.0173 (5)	0.0164 (6)	0.0162 (6)	0.0032 (4)	0.0016 (4)	0.0000 (5)
C2A	0.0183 (5)	0.0164 (6)	0.0195 (6)	0.0013 (4)	0.0029 (5)	0.0007 (5)
C3A	0.0162 (5)	0.0272 (7)	0.0160 (6)	0.0042 (5)	−0.0005 (5)	−0.0044 (5)
C4A	0.0223 (6)	0.0225 (7)	0.0167 (6)	0.0069 (5)	0.0033 (5)	0.0039 (5)
C5A	0.0236 (6)	0.0162 (6)	0.0234 (6)	0.0039 (5)	0.0032 (5)	0.0030 (5)
C6A	0.0195 (6)	0.0156 (6)	0.0200 (6)	0.0030 (4)	−0.0003 (5)	−0.0010 (5)
C7A	0.0299 (7)	0.0157 (6)	0.0237 (7)	−0.0018 (5)	−0.0039 (5)	−0.0019 (5)
C8A	0.0281 (7)	0.0219 (7)	0.0329 (8)	−0.0043 (5)	−0.0065 (6)	0.0024 (6)
C9A	0.0229 (6)	0.0211 (7)	0.0180 (6)	0.0006 (5)	−0.0047 (5)	0.0009 (5)
C10A	0.0201 (6)	0.0334 (8)	0.0243 (7)	0.0004 (5)	−0.0031 (5)	0.0007 (6)
C11A	0.0204 (6)	0.0353 (8)	0.0268 (7)	0.0071 (5)	−0.0003 (5)	−0.0043 (6)
C12A	0.0311 (7)	0.0250 (7)	0.0198 (6)	0.0099 (5)	0.0036 (5)	0.0014 (5)
C13A	0.0229 (6)	0.0200 (7)	0.0170 (6)	0.0023 (5)	0.0043 (5)	0.0011 (5)
C14A	0.0200 (6)	0.0190 (6)	0.0115 (5)	0.0017 (4)	−0.0003 (4)	0.0007 (4)
C15A	0.0216 (6)	0.0156 (6)	0.0197 (6)	0.0008 (5)	0.0048 (5)	0.0028 (5)
C16A	0.0187 (6)	0.0174 (6)	0.0196 (6)	0.0006 (4)	0.0024 (5)	0.0009 (5)
C17A	0.0186 (6)	0.0208 (6)	0.0188 (6)	0.0058 (5)	0.0007 (5)	0.0010 (5)
C18A	0.0209 (6)	0.0325 (8)	0.0274 (7)	0.0048 (5)	0.0054 (5)	0.0055 (6)
C19A	0.0344 (7)	0.0273 (7)	0.0156 (6)	0.0134 (6)	−0.0047 (5)	−0.0041 (5)
Cl2	0.0620 (3)	0.0449 (2)	0.01714 (16)	0.02406 (19)	−0.00841 (16)	−0.00375 (15)
N1B	0.0146 (4)	0.0161 (5)	0.0179 (5)	0.0004 (4)	0.0016 (4)	0.0033 (4)
N2B	0.0232 (5)	0.0235 (6)	0.0120 (5)	0.0056 (4)	−0.0003 (4)	0.0028 (4)
C1B	0.0195 (6)	0.0161 (6)	0.0194 (6)	0.0061 (4)	0.0060 (5)	0.0030 (5)
C2B	0.0233 (6)	0.0197 (6)	0.0180 (6)	0.0090 (5)	0.0051 (5)	0.0027 (5)
C3B	0.0352 (7)	0.0300 (8)	0.0170 (6)	0.0187 (6)	0.0037 (5)	0.0016 (5)
C4B	0.0474 (9)	0.0335 (8)	0.0230 (7)	0.0240 (7)	0.0169 (7)	0.0138 (6)
C5B	0.0317 (7)	0.0229 (7)	0.0403 (9)	0.0096 (6)	0.0197 (7)	0.0139 (6)
C6B	0.0230 (6)	0.0173 (7)	0.0336 (8)	0.0052 (5)	0.0100 (6)	0.0055 (6)
C7B	0.0254 (7)	0.0231 (8)	0.0546 (10)	−0.0053 (6)	0.0054 (7)	0.0073 (7)
C8B	0.0202 (6)	0.0238 (8)	0.0424 (9)	−0.0036 (5)	0.0005 (6)	−0.0068 (6)
C9B	0.0183 (6)	0.0244 (7)	0.0235 (7)	0.0012 (5)	0.0000 (5)	−0.0034 (5)
C10B	0.0185 (6)	0.0394 (9)	0.0258 (7)	0.0027 (5)	−0.0026 (5)	−0.0026 (6)
C11B	0.0208 (6)	0.0433 (9)	0.0234 (7)	0.0107 (6)	−0.0020 (5)	0.0014 (6)
C12B	0.0300 (7)	0.0271 (7)	0.0161 (6)	0.0115 (5)	0.0018 (5)	0.0034 (5)
C13B	0.0199 (6)	0.0218 (7)	0.0127 (5)	0.0037 (5)	0.0005 (4)	0.0022 (5)
C14B	0.0165 (5)	0.0216 (6)	0.0146 (5)	0.0028 (4)	0.0003 (4)	0.0002 (5)
C15B	0.0146 (5)	0.0160 (6)	0.0163 (6)	−0.0003 (4)	−0.0001 (4)	0.0005 (4)
C16B	0.0192 (6)	0.0208 (7)	0.0179 (6)	0.0043 (5)	0.0026 (5)	0.0052 (5)
C17B	0.0185 (6)	0.0280 (7)	0.0147 (6)	0.0067 (5)	−0.0012 (5)	0.0026 (5)
C18B	0.0202 (6)	0.0338 (8)	0.0209 (6)	0.0079 (5)	0.0033 (5)	0.0085 (6)
C19B	0.0419 (8)	0.0279 (8)	0.0187 (6)	0.0086 (6)	−0.0052 (6)	−0.0038 (5)
O1C	0.0265 (5)	0.0232 (5)	0.0175 (4)	0.0002 (4)	−0.0041 (4)	0.0073 (4)
O21C	0.0333 (5)	0.0177 (5)	0.0361 (6)	−0.0010 (4)	−0.0094 (4)	0.0087 (4)
O22C	0.0277 (5)	0.0283 (6)	0.0505 (7)	−0.0049 (4)	−0.0219 (5)	0.0079 (5)
O41C	0.0491 (6)	0.0320 (6)	0.0153 (5)	0.0098 (5)	−0.0084 (4)	0.0002 (4)
O42C	0.0370 (6)	0.0373 (6)	0.0268 (5)	−0.0023 (5)	0.0037 (4)	0.0141 (5)
O61C	0.0588 (7)	0.0406 (7)	0.0383 (7)	−0.0310 (6)	−0.0217 (6)	0.0118 (6)
O62C	0.0384 (6)	0.0463 (7)	0.0242 (5)	−0.0086 (5)	−0.0122 (5)	0.0095 (5)
N2C	0.0214 (5)	0.0155 (5)	0.0260 (6)	0.0004 (4)	−0.0055 (4)	0.0011 (4)
N4C	0.0302 (6)	0.0240 (6)	0.0162 (5)	0.0088 (5)	0.0026 (4)	0.0034 (4)
N6C	0.0234 (5)	0.0232 (6)	0.0186 (5)	−0.0008 (4)	−0.0040 (4)	−0.0011 (4)
C1C	0.0176 (5)	0.0139 (6)	0.0155 (6)	0.0035 (4)	−0.0023 (4)	0.0005 (4)
C2C	0.0165 (5)	0.0124 (6)	0.0191 (6)	0.0019 (4)	−0.0026 (4)	0.0002 (4)
C3C	0.0199 (6)	0.0164 (6)	0.0168 (6)	0.0070 (4)	−0.0050 (5)	−0.0025 (5)
C4C	0.0219 (6)	0.0176 (6)	0.0129 (5)	0.0066 (5)	0.0006 (4)	0.0023 (4)
C5C	0.0174 (5)	0.0161 (6)	0.0186 (6)	0.0019 (4)	0.0009 (5)	0.0006 (5)
C6C	0.0177 (5)	0.0166 (6)	0.0158 (6)	0.0026 (4)	−0.0039 (4)	−0.0011 (4)
O1D	0.0234 (4)	0.0242 (5)	0.0174 (4)	−0.0037 (4)	−0.0030 (4)	0.0060 (4)
O21D	0.0484 (6)	0.0392 (7)	0.0202 (5)	−0.0180 (5)	−0.0144 (5)	0.0097 (5)
O22D	0.0331 (5)	0.0403 (6)	0.0244 (5)	−0.0216 (4)	−0.0022 (4)	−0.0009 (5)
O41D	0.0382 (6)	0.0271 (6)	0.0280 (5)	0.0007 (4)	0.0044 (4)	0.0116 (4)
O42D	0.0480 (6)	0.0337 (6)	0.0167 (5)	0.0121 (5)	−0.0057 (4)	0.0041 (4)
O61D	0.0492 (7)	0.0487 (8)	0.0557 (8)	−0.0222 (6)	−0.0348 (6)	0.0102 (6)
O62D	0.0456 (6)	0.0176 (5)	0.0339 (6)	−0.0047 (4)	−0.0033 (5)	0.0010 (4)
N2D	0.0169 (5)	0.0190 (5)	0.0158 (5)	0.0004 (4)	−0.0005 (4)	−0.0022 (4)
N4D	0.0316 (6)	0.0240 (6)	0.0163 (5)	0.0104 (5)	0.0027 (5)	0.0046 (4)
N6D	0.0288 (6)	0.0232 (6)	0.0230 (6)	−0.0071 (5)	−0.0044 (5)	−0.0022 (5)
C1D	0.0153 (5)	0.0146 (6)	0.0150 (5)	0.0034 (4)	−0.0020 (4)	0.0001 (4)
C2D	0.0167 (5)	0.0147 (6)	0.0139 (5)	0.0022 (4)	−0.0019 (4)	−0.0020 (4)
C3D	0.0185 (5)	0.0131 (6)	0.0183 (6)	0.0022 (4)	0.0026 (5)	0.0007 (5)
C4D	0.0229 (6)	0.0170 (6)	0.0140 (5)	0.0061 (5)	0.0010 (5)	0.0036 (5)
C5D	0.0221 (6)	0.0217 (7)	0.0160 (6)	0.0055 (5)	−0.0047 (5)	−0.0023 (5)
C6D	0.0188 (5)	0.0149 (6)	0.0174 (6)	−0.0005 (4)	−0.0025 (5)	−0.0006 (5)

### Geometric parameters (Å, °)

**Table:**

Cl1—C3A	1.7427 (13)	C7B—C8B	1.528 (2)
N1A—C1A	1.4329 (15)	C7B—H7BA	0.9900
N1A—C14A	1.4441 (15)	C7B—H7BB	0.9900
N1A—C15A	1.4656 (15)	C8B—C9B	1.496 (2)
N2A—C19A	1.4879 (16)	C8B—H8BA	0.9900
N2A—C18A	1.4879 (16)	C8B—H8BB	0.9900
N2A—C17A	1.4940 (15)	C9B—C10B	1.3979 (18)
N2A—H2AB	0.9300	C9B—C14B	1.3985 (17)
C1A—C2A	1.3912 (17)	C10B—C11B	1.387 (2)
C1A—C6A	1.4050 (17)	C10B—H10B	0.9500
C2A—C3A	1.3870 (17)	C11B—C12B	1.381 (2)
C2A—H2AA	0.9500	C11B—H11B	0.9500
C3A—C4A	1.3841 (18)	C12B—C13B	1.3935 (17)
C4A—C5A	1.3950 (18)	C12B—H12B	0.9500
C4A—H4AA	0.9500	C13B—C14B	1.3959 (18)
C5A—C6A	1.3890 (17)	C13B—H13B	0.9500
C5A—H5AA	0.9500	C15B—C16B	1.5238 (17)
C6A—C7A	1.5019 (17)	C15B—H15C	0.9900
C7A—C8A	1.5214 (19)	C15B—H15D	0.9900
C7A—H7AA	0.9900	C16B—C17B	1.5117 (19)
C7A—H7AB	0.9900	C16B—H16C	0.9900
C8A—C9A	1.5145 (19)	C16B—H16D	0.9900
C8A—H8AA	0.9900	C17B—H17C	0.9900
C8A—H8AB	0.9900	C17B—H17D	0.9900
C9A—C10A	1.3983 (19)	C18B—H18D	0.9800
C9A—C14A	1.4043 (17)	C18B—H18E	0.9800
C10A—C11A	1.387 (2)	C18B—H18F	0.9800
C10A—H10A	0.9500	C19B—H19D	0.9800
C11A—C12A	1.3765 (19)	C19B—H19E	0.9800
C11A—H11A	0.9500	C19B—H19F	0.9800
C12A—C13A	1.3887 (18)	O1C—C1C	1.2474 (14)
C12A—H12A	0.9500	O21C—N2C	1.2265 (13)
C13A—C14A	1.3984 (18)	O22C—N2C	1.2316 (13)
C13A—H13A	0.9500	O41C—N4C	1.2343 (14)
C15A—C16A	1.5273 (17)	O42C—N4C	1.2326 (14)
C15A—H15A	0.9900	O61C—N6C	1.2221 (14)
C15A—H15B	0.9900	O62C—N6C	1.2248 (14)
C16A—C17A	1.5182 (18)	N2C—C2C	1.4606 (15)
C16A—H16A	0.9900	N4C—C4C	1.4447 (15)
C16A—H16B	0.9900	N6C—C6C	1.4501 (15)
C17A—H17A	0.9900	C1C—C2C	1.4540 (16)
C17A—H17B	0.9900	C1C—C6C	1.4558 (16)
C18A—H18A	0.9800	C2C—C3C	1.3678 (17)
C18A—H18B	0.9800	C3C—C4C	1.3917 (17)
C18A—H18C	0.9800	C3C—H3CA	0.9500
C19A—H19A	0.9800	C4C—C5C	1.3836 (16)
C19A—H19B	0.9800	C5C—C6C	1.3773 (16)
C19A—H19C	0.9800	C5C—H5CA	0.9500
Cl2—C3B	1.7459 (15)	O1D—C1D	1.2429 (14)
N1B—C1B	1.4240 (15)	O21D—N2D	1.2234 (13)
N1B—C14B	1.4435 (15)	O22D—N2D	1.2218 (13)
N1B—C15B	1.4692 (14)	O41D—N4D	1.2377 (14)
N2B—C18B	1.4920 (16)	O42D—N4D	1.2323 (14)
N2B—C19B	1.4950 (17)	O61D—N6D	1.2244 (14)
N2B—C17B	1.5016 (16)	O62D—N6D	1.2236 (15)
N2B—H2BB	0.9300	N2D—C2D	1.4474 (14)
C1B—C2B	1.4004 (17)	N4D—C4D	1.4491 (15)
C1B—C6B	1.4150 (17)	N6D—C6D	1.4602 (16)
C2B—C3B	1.3864 (17)	C1D—C6D	1.4534 (16)
C2B—H2BA	0.9500	C1D—C2D	1.4552 (16)
C3B—C4B	1.381 (2)	C2D—C3D	1.3789 (16)
C4B—C5B	1.379 (2)	C3D—C4D	1.3800 (17)
C4B—H4BA	0.9500	C3D—H3DA	0.9500
C5B—C6B	1.402 (2)	C4D—C5D	1.3913 (18)
C5B—H5BA	0.9500	C5D—C6D	1.3703 (17)
C6B—C7B	1.515 (2)	C5D—H5DA	0.9500
			
C1A—N1A—C14A	114.64 (10)	C6B—C7B—C8B	118.55 (11)
C1A—N1A—C15A	116.51 (10)	C6B—C7B—H7BA	107.7
C14A—N1A—C15A	116.45 (10)	C8B—C7B—H7BA	107.7
C19A—N2A—C18A	110.55 (10)	C6B—C7B—H7BB	107.7
C19A—N2A—C17A	110.57 (9)	C8B—C7B—H7BB	107.7
C18A—N2A—C17A	112.47 (10)	H7BA—C7B—H7BB	107.1
C19A—N2A—H2AB	107.7	C9B—C8B—C7B	110.77 (13)
C18A—N2A—H2AB	107.7	C9B—C8B—H8BA	109.5
C17A—N2A—H2AB	107.7	C7B—C8B—H8BA	109.5
C2A—C1A—C6A	120.33 (11)	C9B—C8B—H8BB	109.5
C2A—C1A—N1A	122.10 (11)	C7B—C8B—H8BB	109.5
C6A—C1A—N1A	117.57 (11)	H8BA—C8B—H8BB	108.1
C3A—C2A—C1A	119.05 (11)	C10B—C9B—C14B	118.76 (13)
C3A—C2A—H2AA	120.5	C10B—C9B—C8B	122.75 (12)
C1A—C2A—H2AA	120.5	C14B—C9B—C8B	118.35 (11)
C4A—C3A—C2A	122.16 (11)	C11B—C10B—C9B	120.80 (13)
C4A—C3A—Cl1	118.28 (10)	C11B—C10B—H10B	119.6
C2A—C3A—Cl1	119.53 (10)	C9B—C10B—H10B	119.6
C3A—C4A—C5A	117.92 (11)	C12B—C11B—C10B	119.94 (12)
C3A—C4A—H4AA	121.0	C12B—C11B—H11B	120.0
C5A—C4A—H4AA	121.0	C10B—C11B—H11B	120.0
C6A—C5A—C4A	121.75 (12)	C11B—C12B—C13B	120.47 (13)
C6A—C5A—H5AA	119.1	C11B—C12B—H12B	119.8
C4A—C5A—H5AA	119.1	C13B—C12B—H12B	119.8
C5A—C6A—C1A	118.74 (11)	C12B—C13B—C14B	119.50 (12)
C5A—C6A—C7A	122.46 (11)	C12B—C13B—H13B	120.2
C1A—C6A—C7A	118.80 (11)	C14B—C13B—H13B	120.2
C6A—C7A—C8A	112.37 (12)	C13B—C14B—C9B	120.51 (11)
C6A—C7A—H7AA	109.1	C13B—C14B—N1B	122.00 (11)
C8A—C7A—H7AA	109.1	C9B—C14B—N1B	117.48 (11)
C6A—C7A—H7AB	109.1	N1B—C15B—C16B	111.24 (9)
C8A—C7A—H7AB	109.1	N1B—C15B—H15C	109.4
H7AA—C7A—H7AB	107.9	C16B—C15B—H15C	109.4
C9A—C8A—C7A	118.47 (11)	N1B—C15B—H15D	109.4
C9A—C8A—H8AA	107.7	C16B—C15B—H15D	109.4
C7A—C8A—H8AA	107.7	H15C—C15B—H15D	108.0
C9A—C8A—H8AB	107.7	C17B—C16B—C15B	115.30 (10)
C7A—C8A—H8AB	107.7	C17B—C16B—H16C	108.4
H8AA—C8A—H8AB	107.1	C15B—C16B—H16C	108.5
C10A—C9A—C14A	117.66 (12)	C17B—C16B—H16D	108.5
C10A—C9A—C8A	115.25 (12)	C15B—C16B—H16D	108.4
C14A—C9A—C8A	127.09 (12)	H16C—C16B—H16D	107.5
C11A—C10A—C9A	122.66 (13)	N2B—C17B—C16B	114.80 (10)
C11A—C10A—H10A	118.7	N2B—C17B—H17C	108.6
C9A—C10A—H10A	118.7	C16B—C17B—H17C	108.6
C12A—C11A—C10A	119.06 (12)	N2B—C17B—H17D	108.6
C12A—C11A—H11A	120.5	C16B—C17B—H17D	108.6
C10A—C11A—H11A	120.5	H17C—C17B—H17D	107.5
C11A—C12A—C13A	119.82 (13)	N2B—C18B—H18D	109.5
C11A—C12A—H12A	120.1	N2B—C18B—H18E	109.5
C13A—C12A—H12A	120.1	H18D—C18B—H18E	109.5
C12A—C13A—C14A	121.31 (12)	N2B—C18B—H18F	109.5
C12A—C13A—H13A	119.3	H18D—C18B—H18F	109.5
C14A—C13A—H13A	119.3	H18E—C18B—H18F	109.5
C13A—C14A—C9A	119.45 (11)	N2B—C19B—H19D	109.5
C13A—C14A—N1A	119.22 (11)	N2B—C19B—H19E	109.5
C9A—C14A—N1A	121.33 (11)	H19D—C19B—H19E	109.5
N1A—C15A—C16A	111.90 (10)	N2B—C19B—H19F	109.5
N1A—C15A—H15A	109.2	H19D—C19B—H19F	109.5
C16A—C15A—H15A	109.2	H19E—C19B—H19F	109.5
N1A—C15A—H15B	109.2	O21C—N2C—O22C	123.02 (10)
C16A—C15A—H15B	109.2	O21C—N2C—C2C	118.88 (10)
H15A—C15A—H15B	107.9	O22C—N2C—C2C	118.01 (10)
C17A—C16A—C15A	110.88 (10)	O42C—N4C—O41C	123.09 (11)
C17A—C16A—H16A	109.5	O42C—N4C—C4C	118.58 (11)
C15A—C16A—H16A	109.5	O41C—N4C—C4C	118.33 (11)
C17A—C16A—H16B	109.5	O61C—N6C—O62C	121.82 (11)
C15A—C16A—H16B	109.5	O61C—N6C—C6C	118.33 (11)
H16A—C16A—H16B	108.1	O62C—N6C—C6C	119.83 (10)
N2A—C17A—C16A	113.17 (10)	O1C—C1C—C2C	122.71 (10)
N2A—C17A—H17A	108.9	O1C—C1C—C6C	125.28 (11)
C16A—C17A—H17A	108.9	C2C—C1C—C6C	111.96 (10)
N2A—C17A—H17B	108.9	C3C—C2C—C1C	124.71 (11)
C16A—C17A—H17B	108.9	C3C—C2C—N2C	116.39 (10)
H17A—C17A—H17B	107.8	C1C—C2C—N2C	118.89 (10)
N2A—C18A—H18A	109.5	C2C—C3C—C4C	118.73 (11)
N2A—C18A—H18B	109.5	C2C—C3C—H3CA	120.6
H18A—C18A—H18B	109.5	C4C—C3C—H3CA	120.6
N2A—C18A—H18C	109.5	C5C—C4C—C3C	121.31 (11)
H18A—C18A—H18C	109.5	C5C—C4C—N4C	119.49 (11)
H18B—C18A—H18C	109.5	C3C—C4C—N4C	119.14 (11)
N2A—C19A—H19A	109.5	C6C—C5C—C4C	119.64 (11)
N2A—C19A—H19B	109.5	C6C—C5C—H5CA	120.2
H19A—C19A—H19B	109.5	C4C—C5C—H5CA	120.2
N2A—C19A—H19C	109.5	C5C—C6C—N6C	116.65 (10)
H19A—C19A—H19C	109.5	C5C—C6C—C1C	123.51 (11)
H19B—C19A—H19C	109.5	N6C—C6C—C1C	119.84 (10)
C1B—N1B—C14B	113.91 (9)	O22D—N2D—O21D	121.40 (10)
C1B—N1B—C15B	116.48 (9)	O22D—N2D—C2D	118.52 (10)
C14B—N1B—C15B	116.06 (9)	O21D—N2D—C2D	120.08 (10)
C18B—N2B—C19B	110.68 (11)	O42D—N4D—O41D	123.15 (11)
C18B—N2B—C17B	112.88 (10)	O42D—N4D—C4D	118.23 (11)
C19B—N2B—C17B	109.41 (10)	O41D—N4D—C4D	118.62 (11)
C18B—N2B—H2BB	107.9	O62D—N6D—O61D	123.40 (12)
C19B—N2B—H2BB	107.9	O62D—N6D—C6D	118.39 (11)
C17B—N2B—H2BB	107.9	O61D—N6D—C6D	118.16 (11)
C2B—C1B—C6B	119.35 (11)	O1D—C1D—C6D	122.60 (11)
C2B—C1B—N1B	119.55 (10)	O1D—C1D—C2D	126.13 (11)
C6B—C1B—N1B	121.08 (11)	C6D—C1D—C2D	111.22 (10)
C3B—C2B—C1B	120.54 (12)	C3D—C2D—N2D	116.41 (10)
C3B—C2B—H2BA	119.7	C3D—C2D—C1D	123.81 (10)
C1B—C2B—H2BA	119.7	N2D—C2D—C1D	119.79 (10)
C4B—C3B—C2B	121.28 (13)	C2D—C3D—C4D	119.71 (11)
C4B—C3B—Cl2	119.96 (11)	C2D—C3D—H3DA	120.1
C2B—C3B—Cl2	118.74 (11)	C4D—C3D—H3DA	120.1
C5B—C4B—C3B	117.97 (13)	C3D—C4D—C5D	121.16 (11)
C5B—C4B—H4BA	121.0	C3D—C4D—N4D	119.45 (11)
C3B—C4B—H4BA	121.0	C5D—C4D—N4D	119.37 (11)
C4B—C5B—C6B	123.43 (13)	C6D—C5D—C4D	118.51 (11)
C4B—C5B—H5BA	118.3	C6D—C5D—H5DA	120.7
C6B—C5B—H5BA	118.3	C4D—C5D—H5DA	120.7
C5B—C6B—C1B	117.41 (13)	C5D—C6D—C1D	125.15 (11)
C5B—C6B—C7B	116.14 (12)	C5D—C6D—N6D	117.09 (11)
C1B—C6B—C7B	126.43 (12)	C1D—C6D—N6D	117.76 (10)
			
C14A—N1A—C1A—C2A	−107.63 (13)	C12B—C13B—C14B—N1B	−177.79 (11)
C15A—N1A—C1A—C2A	33.30 (16)	C10B—C9B—C14B—C13B	−1.22 (19)
C14A—N1A—C1A—C6A	72.36 (14)	C8B—C9B—C14B—C13B	−176.97 (12)
C15A—N1A—C1A—C6A	−146.71 (11)	C10B—C9B—C14B—N1B	178.02 (11)
C6A—C1A—C2A—C3A	−1.34 (18)	C8B—C9B—C14B—N1B	2.26 (18)
N1A—C1A—C2A—C3A	178.65 (11)	C1B—N1B—C14B—C13B	102.50 (13)
C1A—C2A—C3A—C4A	−0.64 (19)	C15B—N1B—C14B—C13B	−36.93 (16)
C1A—C2A—C3A—Cl1	−178.58 (9)	C1B—N1B—C14B—C9B	−76.72 (14)
C2A—C3A—C4A—C5A	1.07 (18)	C15B—N1B—C14B—C9B	143.84 (12)
Cl1—C3A—C4A—C5A	179.03 (9)	C1B—N1B—C15B—C16B	165.18 (10)
C3A—C4A—C5A—C6A	0.48 (19)	C14B—N1B—C15B—C16B	−56.43 (14)
C4A—C5A—C6A—C1A	−2.38 (19)	N1B—C15B—C16B—C17B	−53.28 (14)
C4A—C5A—C6A—C7A	177.53 (12)	C18B—N2B—C17B—C16B	−54.25 (14)
C2A—C1A—C6A—C5A	2.80 (18)	C19B—N2B—C17B—C16B	−177.97 (11)
N1A—C1A—C6A—C5A	−177.19 (11)	C15B—C16B—C17B—N2B	−59.81 (14)
C2A—C1A—C6A—C7A	−177.11 (11)	O1C—C1C—C2C—C3C	−172.94 (12)
N1A—C1A—C6A—C7A	2.90 (17)	C6C—C1C—C2C—C3C	4.51 (17)
C5A—C6A—C7A—C8A	108.32 (14)	O1C—C1C—C2C—N2C	8.32 (18)
C1A—C6A—C7A—C8A	−71.77 (15)	C6C—C1C—C2C—N2C	−174.22 (10)
C6A—C7A—C8A—C9A	53.41 (17)	O21C—N2C—C2C—C3C	−146.36 (12)
C7A—C8A—C9A—C10A	−178.17 (13)	O22C—N2C—C2C—C3C	30.17 (17)
C7A—C8A—C9A—C14A	1.9 (2)	O21C—N2C—C2C—C1C	32.47 (17)
C14A—C9A—C10A—C11A	1.1 (2)	O22C—N2C—C2C—C1C	−151.00 (12)
C8A—C9A—C10A—C11A	−178.89 (13)	C1C—C2C—C3C—C4C	−2.84 (19)
C9A—C10A—C11A—C12A	−1.9 (2)	N2C—C2C—C3C—C4C	175.92 (11)
C10A—C11A—C12A—C13A	0.9 (2)	C2C—C3C—C4C—C5C	0.02 (18)
C11A—C12A—C13A—C14A	0.7 (2)	C2C—C3C—C4C—N4C	−177.33 (11)
C12A—C13A—C14A—C9A	−1.53 (19)	O42C—N4C—C4C—C5C	4.18 (18)
C12A—C13A—C14A—N1A	179.06 (11)	O41C—N4C—C4C—C5C	−176.14 (11)
C10A—C9A—C14A—C13A	0.63 (18)	O42C—N4C—C4C—C3C	−178.43 (11)
C8A—C9A—C14A—C13A	−179.43 (13)	O41C—N4C—C4C—C3C	1.26 (17)
C10A—C9A—C14A—N1A	−179.98 (12)	C3C—C4C—C5C—C6C	0.60 (18)
C8A—C9A—C14A—N1A	0.0 (2)	N4C—C4C—C5C—C6C	177.94 (11)
C1A—N1A—C14A—C13A	119.29 (12)	C4C—C5C—C6C—N6C	−178.20 (11)
C15A—N1A—C14A—C13A	−21.67 (16)	C4C—C5C—C6C—C1C	1.51 (19)
C1A—N1A—C14A—C9A	−60.10 (15)	O61C—N6C—C6C—C5C	−10.99 (18)
C15A—N1A—C14A—C9A	158.94 (11)	O62C—N6C—C6C—C5C	167.41 (12)
C1A—N1A—C15A—C16A	59.19 (14)	O61C—N6C—C6C—C1C	169.29 (13)
C14A—N1A—C15A—C16A	−160.59 (10)	O62C—N6C—C6C—C1C	−12.31 (18)
N1A—C15A—C16A—C17A	63.99 (13)	O1C—C1C—C6C—C5C	173.58 (12)
C19A—N2A—C17A—C16A	−178.29 (10)	C2C—C1C—C6C—C5C	−3.79 (17)
C18A—N2A—C17A—C16A	−54.17 (13)	O1C—C1C—C6C—N6C	−6.71 (19)
C15A—C16A—C17A—N2A	−168.79 (10)	C2C—C1C—C6C—N6C	175.92 (10)
C14B—N1B—C1B—C2B	−122.02 (12)	O22D—N2D—C2D—C3D	0.77 (16)
C15B—N1B—C1B—C2B	17.24 (16)	O21D—N2D—C2D—C3D	−179.14 (12)
C14B—N1B—C1B—C6B	56.77 (16)	O22D—N2D—C2D—C1D	−179.04 (11)
C15B—N1B—C1B—C6B	−163.97 (11)	O21D—N2D—C2D—C1D	1.04 (17)
C6B—C1B—C2B—C3B	−1.29 (18)	O1D—C1D—C2D—C3D	171.41 (12)
N1B—C1B—C2B—C3B	177.51 (11)	C6D—C1D—C2D—C3D	−6.16 (16)
C1B—C2B—C3B—C4B	0.2 (2)	O1D—C1D—C2D—N2D	−8.79 (18)
C1B—C2B—C3B—Cl2	−178.11 (9)	C6D—C1D—C2D—N2D	173.63 (10)
C2B—C3B—C4B—C5B	0.6 (2)	N2D—C2D—C3D—C4D	−178.30 (11)
Cl2—C3B—C4B—C5B	178.84 (10)	C1D—C2D—C3D—C4D	1.50 (18)
C3B—C4B—C5B—C6B	−0.2 (2)	C2D—C3D—C4D—C5D	2.70 (18)
C4B—C5B—C6B—C1B	−0.9 (2)	C2D—C3D—C4D—N4D	−178.33 (11)
C4B—C5B—C6B—C7B	177.65 (13)	O42D—N4D—C4D—C3D	174.21 (11)
C2B—C1B—C6B—C5B	1.62 (18)	O41D—N4D—C4D—C3D	−5.20 (17)
N1B—C1B—C6B—C5B	−177.17 (11)	O42D—N4D—C4D—C5D	−6.80 (17)
C2B—C1B—C6B—C7B	−176.77 (13)	O41D—N4D—C4D—C5D	173.79 (12)
N1B—C1B—C6B—C7B	4.4 (2)	C3D—C4D—C5D—C6D	−1.40 (19)
C5B—C6B—C7B—C8B	179.37 (13)	N4D—C4D—C5D—C6D	179.63 (11)
C1B—C6B—C7B—C8B	−2.2 (2)	C4D—C5D—C6D—C1D	−4.27 (19)
C6B—C7B—C8B—C9B	−56.27 (17)	C4D—C5D—C6D—N6D	176.05 (11)
C7B—C8B—C9B—C10B	−105.22 (15)	O1D—C1D—C6D—C5D	−170.03 (12)
C7B—C8B—C9B—C14B	70.35 (16)	C2D—C1D—C6D—C5D	7.64 (17)
C14B—C9B—C10B—C11B	0.6 (2)	O1D—C1D—C6D—N6D	9.64 (18)
C8B—C9B—C10B—C11B	176.18 (14)	C2D—C1D—C6D—N6D	−172.68 (10)
C9B—C10B—C11B—C12B	−0.2 (2)	O62D—N6D—C6D—C5D	−146.57 (13)
C10B—C11B—C12B—C13B	0.4 (2)	O61D—N6D—C6D—C5D	31.08 (19)
C11B—C12B—C13B—C14B	−0.99 (19)	O62D—N6D—C6D—C1D	33.73 (18)
C12B—C13B—C14B—C9B	1.41 (19)	O61D—N6D—C6D—C1D	−148.62 (14)

### Hydrogen-bond geometry (Å, °)

**Table:**

*D*—H···*A*	*D*—H	H···*A*	*D*···*A*	*D*—H···*A*
N2A—H2AB···O1D	0.93	1.85	2.7000 (13)	151
N2A—H2AB···O21D	0.93	2.23	2.8982 (14)	128
N2B—H2BB···O1C	0.93	1.92	2.6970 (13)	140
N2B—H2BB···O62C	0.93	2.36	3.0657 (15)	133
C12A—H12A···*Cg*7^i^	0.95	2.83	3.656 (4)	145

Symmetry codes: (i) *x*, *y*−1, *z*.

### Table 2 π-π hydrogen-bond geometry (Å)

**Table:**

Cg···Cg	D···A
Cg1···Cg14^i^	3.838 (8)
Cg7···Cg13^ii^	3.473 (5)
Cg13···Cg14^i^	3.590 (5)

Symmetry codes: (i) x, y, z; (ii) 1-x, y, z; Cg1, Cg7, Cg13, Cg14 are the centroids of the C1A–C6A, C1B–C6B, C1C–C6C and C1D–C6D rings.

## References

[bb1] Albert, U., Aguglia, E., Maina, G. & Bogetto, F. (2002). *J. Clin. Psychiatry*, **63**, 1004–1009. 10.4088/jcp.v63n1108 12444814

[bb2] Allen, F. H., Johnson, O., Shields, G. P., Smith, B. R. & Towler, M. (2004). *J. Appl. Cryst.* **37**, 335–338.

[bb3] Becke, A. D. (1988). *Phys. Rev. A*, **38**, 3098–3100. 10.1103/physreva.38.3098 9900728

[bb4] Bindya, S., Wong, W.-T., Ashok, M. A., Yathirajan, H. S. & Rathore, R. S. (2007). *Acta Cryst.* C**63**, o546–o548. 10.1107/S0108270107037468 17762129

[bb5] Cassano, G. B., Petracca, A., Perugi, G., Nisita, C., Musetti, L., Mengali, F. & McNair, D. M. (1988). *J. Affect. Disord.* **14**, 123–127. 10.1016/0165-0327(88)90054-7 2966825

[bb6] Daley, E., Wilkie, D., Loesch, A., Hargreaves, I. P., Kendall, D. A., Pilkington, G. J. & Bates, T. E. (2005). *Biochem. Biophys. Res. Commun.* **328**, 623–632. 10.1016/j.bbrc.2005.01.028 15694394

[bb7] Frisch, M. J., *et al.* (2004). *GAUSSIAN03* Gaussian Inc., Wallingford, CT, USA.

[bb8] Guimaraes, F. S., Zuardi, A. W. & Graeff, F. G. (1987). *J. Psychopharmacol.* **1**, 184–192. 10.1177/026988118700100305 22158980

[bb9] Hallberg, A., Hintermeister, N. M., Martin, A. R., Bates, R. B. & Ortega, R. B. (1984). *Acta Cryst.* C**40**, 2110–2112.

[bb10] Harrison, W. T. A., Bindya, S., Ashok, M. A., Yathirajan, H. S. & Narayana, B. (2007). *Acta Cryst.* E**63**, o3143.

[bb11] Harrison, W. T. A., Sreevidya, T. V., Narayana, B., Sarojini, B. K. & Yathirajan, H. S. (2007). *Acta Cryst.* E**63**, o3871.

[bb12] Lee, C., Yang, W. & Parr, R. G. (1988). *Phys. Rev. B*, **37**, 785–789. 10.1103/physrevb.37.785 9944570

[bb13] Macrae, C. F., Edgington, P. R., McCabe, P., Pidcock, E., Shields, G. P., Taylor, R., Towler, M. & van de Streek, J. (2006). *J. Appl. Cryst.* **39**, 453–457.

[bb14] Oxford Diffraction (2007). *CrysAlis PRO* and *CrysAlis RED* Oxford Diffraction Ltd, Abingdon, Oxfordshire, England.

[bb15] Post, M. L. & Horn, A. S. (1977). *Acta Cryst.* B**33**, 2590–2595.

[bb16] Post, M. L., Kennard, O. & Horn, A. S. (1975). *Acta Cryst.* B**31**, 1008–1013.

[bb17] Schmidt, J. R. & Polik, W. F. (2007). *WebMO Pro.* WebMO LLC, Holland, MI, USA, available from http://www.webmo.net.

[bb18] Sheldrick, G. M. (2008). *Acta Cryst.* A**64**, 112–122. 10.1107/S0108767307043930 18156677

[bb19] Spek, A. L. (2009). *Acta Cryst.* D**65**, 148–155. 10.1107/S090744490804362X PMC2631630 19171970

[bb20] Swamy, M. T., Ashok, M. A., Yathirajan, H. S., Narayana, B. & Bolte, M. (2007). *Acta Cryst.* E**63**, o4919.

[bb21] Yathirajan, H. S., Ashok, M. A., Narayana Achar, B. & Bolte, M. (2007). *Acta Cryst.* E**63**, o1691–o1692.

